# MicroRNAs in cancer: from developmental genes in worms to their clinical application in patients

**DOI:** 10.1038/bjc.2015.253

**Published:** 2015-07-09

**Authors:** M Pichler, G A Calin

**Affiliations:** 1 Department of Experimental Therapeutics, The University of Texas, MD Anderson Cancer Center, Houston, TX, USA; 2 Division of Oncology, Medical University of Graz, Austria; 3 The Center for RNA Interference and Non-coding RNAs, The University of Texas, MD Anderson Cancer Center, Houston, TX, USA

**Keywords:** microRNAs, diagnosis, prognosis, treatment, worms, cancer

## Abstract

Several discoveries have paved the way to personalise cancer medicine and a tremendous gain of knowledge in genomics and molecular mechanisms of cancer progression cumulated over the last years. Big stories in biology commonly start in a simple model system. No wonder microRNAs have been identified as regulators of embryonic development in the nematode *Caenorhabditis elegans*. From the first identification in worms to the first-in-man microRNA-based clinical trial in humans, almost 20 years passed. In this review we follow the story of understanding microRNA alterations in cancer, describe recent developments in the microRNA field and critically discuss their potential as diagnostic, prognostic and therapeutics factors in cancer medicine. We will explain the rationale behind the use of microRNAs in cancer diagnosis and prognosis prediction, but also discuss the limitations and pitfalls associated with this. Novel developments of combined microRNA/siRNA pharmacological approaches will be discussed and most recently data about MXR34, the first-tested microRNA drug will be described.

More than 20 years ago, two groups published their seminal work about the involvement of a small RNA sequence (lin-4) in diverse postembryonic developmental events in the nematode *Caenorhabditis elegans* (Lee *et al*, 1993; Wightman *et al*, 1993). Although at that time the term microRNA had not been created (in the first years after this discovery they had been referred to as small temporal RNAs), the group of Victor Ambros already proposed in their pioneering study fundamental principles of what microRNAs are and how this new class of noncoding RNA works. By determining the size of ∼22 nucleotides, and discovering the interaction of lin-4 with sequence complementary elements in a repeated sequence element in the 3′-untranslated region of lin-14 messenger RNA, they established at this time the basis for a new research direction in developmental biology, physiology and medicine. Another groundbreaking work was published by the group of Thomas Tuschl, where they clearly demonstrated a broader role for these small RNAs in biology and their existence in multiple organisms including vertebrates and humans (Lagos-Quintana *et al*, 2001). According to its relatively small size of about 20 nucleotides, this novel RNA species was termed microRNA. At this time the race to determine the biological function of microRNAs in human diseases was initiated. (Lagos-Quintana *et al*, 2001). Two years later, Calin *et al* published for the first time a direct link between microRNAs and human cancer. In their work, Calin *et al.* reported that miR-15 and miR-16 are located at chromosome 13q14, a region frequently deleted in B-cell chronic lymphocytic leukaemia. In more than two third of cases, these microRNA genes are deleted or their expression is downregulated by other events (Calin *et al*, 2002). Since then, many other important experimental and clinical discoveries have been reported by many different groups. For reasons of space restriction in this review, many of these excellent works could not be cited or discussed here. Summarising the main findings of the last 10 years, it clearly came out that microRNAs are differentially expressed between normal and cancer cells, that they are more or less reflecting tissue-specific expression signatures and that microRNAs can either promote (‘oncomiRs') or suppress tumour development and progression, thereby influencing all hallmarks of cancer (depending on the type of cell and tissue context, Figure 1; Calin and Croce, 2006). Besides their comprehensively and well-described intracellular functions, microRNAs have been found as circulating biomolecules in all body fluids (e.g., blood, urine, sputum or stool). Recently published studies propose that microRNAs are not only ‘passively' circulating byproducts, but also exert a role as intercellular messengers by exosome-mediated transfer between different cells in a ‘hormone-like' manner (Cortez *et al*, 2011). In this review, we are trying to discuss representative examples of the most recent and relevant developments of microRNA research in clinical oncology and their current status of applications in cancer patients. Overall, there are three major topics we are addressing: microRNAs in diagnosis, in prognosis and in therapy of cancer.

## MicroRNAs in Cancer Diagnosis

In many if not all cancer patients, the tumour stage at diagnosis of the underlying malignant disease significantly influences risk of recurrence, progression and death. For many types of cancer, there is currently a lack of early detection methods or screening tests, making the issue of early cancer detection a promising field for microRNA-based diagnostics. On the basis of the above-mentioned features of (1) cancer cell and tissue-specific expression profiles and (2) circulation in body fluids, microRNAs exhibit some characteristics for ideal biomarkers. The relatively high chemical stability of microRNAs in fresh or even formalin-fixed tissues and body fluids is another advantage that increases their potential as diagnostic markers in comparison with longer messenger RNAs or long noncoding RNAs (Blondal *et al*, 2013). As microRNAs are released by healthy and cancerous cells, many attempts have been made to determine the meaning of the specific expression signatures as potential blood-, urine- or stool-based diagnostic markers. Especially in cancers where other early detection methods are not available, expensive or even harmful for patients, microRNA-based biomarkers might possess chances to get established in routine clinical practice. One important example is the potential of microRNAs in lung cancer early-stage detection. Previous studies have clearly indicated that by using low-dose computed tomography (CT)-based screening strategies in high-risk populations, higher detection rates of early-stage lung cancer, results in improved survival rates of patients (Aberle *et al*, 2011). However, owing to the associated relatively high costs and the risk of induction of secondary cancers by (even low) radiation exposure, the widespread clinical application of CT-based screening programs is controversially discussed. In this clinical setting, Montani and colleagues recently published data of a comprehensive large-scale validation study (*n*=1115) of a serum-based microRNA signature (‘miR-Test'). The authors tested this microRNA signature in high-risk individuals enrolled in the Continuous Observation of Smoking Subjects lung cancer screening programme. Of note, the overall accuracy, sensitivity and specificity of this microRNA assay were 74.9% (95% confidence interval (CI)=72.2–77.6%), 77.8% (95% CI=64.2–91.4%) and 74.8% (95% CI=72.1–77.5%), respectively. This study results are striking, as the authors used a well-designed cohort with high number of patients, both supporting meaningfully statistical conclusion (Montani *et al*, 2015). In context of lung cancer detection methods, another recently published study presented data about a sputum-based microRNA panel to identify lung cancer in indeterminate solitary lung nodules. In their study, Xing *et al.* used a training set to develop a biomarker panel of miR-21, -31 and -210, and validated this panel in larger independent sets of samples. Sensitivity and specificity ranged between 80 and 88% in all tested cohorts (Xing *et al*, 2015). In addition to the application of early cancer detection, another promising field of microRNA-based assays in cancer diagnosis is reasoned on the discovery that microRNA expression profiles are highly tissue and cell type specific, allowing the reliable classification of different types of cancer based on the microRNA profile (Rosenfeld *et al*, 2008).

This tissue specificity carries great potential for the diagnosis of cancer of unknown primary origin (CUP) or uncertain origin. Cancer of unknown primary origin alone constitutes 3–5% (50 000–70 000 cases) of all newly diagnosed cancers per year in the United States. Cancer of unknown primary origin presents a clinical challenge as the origin of tumour tissue is crucial for selecting proper treatment plan. Meiri and colleagues published the development and validation of a second-generation microRNA-based custom array that can assign CUP to 42 different types of cancer. In their study, the authors showed an impressive overall assay sensitivity of 85% in a large cohort of 509 CUP samples (Meiri *et al*, 2012). The sensitivity reached 90% for cases in which the assay reported a single answer (>80% of cases). Beyond the use of microRNAs for the classification of CUP, a series of other studies reported about the potential for discrimination of histological subtypes in certain organs. The rationale for developing microRNA assays for differentiating histological subtypes comes from limitations of current diagnostic standards: Low amounts of collected cancer cells by biopsy or only cytological smears make the microscopic diagnosis difficult in some cases and significantly increase the interobserver variability. For instance, Lebanony *et al* (2009) reported about a high-discrimination rate (sensitivity of 96% and specificity of 90%) for miR-205 to identify squamous cell carcinoma of the lung. Over the years, more advanced diagnostic microRNA assays have been developed. In one study, the authors propose a novel diagnostic microRNA-based assay (miRview lung, Rosetta Genomics Ltd.), which can differentiate between the four main types of lung cancer: squamous cell carcinoma of the lung, nonsquamous nonsmall cell lung cancer, carcinoid tumours and small cell carcinoma. On several hundred samples, this assay returned a result for >90% of the samples with overall accuracy of 94% (95% CI, 91–96%), with similar performance observed in pathologic and cytological samples (Gilad *et al*, 2012). Despite impressive results, the true value of these assays has to be interpreted by integrating considerations of the current practice in lung cancer management. On the basis of the approval of patient-tailored drugs (i.e., EGFR inhibitors and ALK inhibitors), the determination of the general mutational landscape in lung cancer tissue is getting more important than only the histological diagnosis, as the mutational spectrum directly and significantly influences the treatment plan. There is an ongoing discussion and also already initiated clinical trials (‘basket' and ‘umbrella' clinical trial design) that aim on histology-independent and aberration-specific clinical trials (Menis *et al*, 2014). The ability to classify histological subtypes by microRNA-based assays has also been successfully demonstrated for kidney cancer, pleura mesothelioma and other types of cancer (Benjamin *et al*, 2010; Spector *et al*, 2013).

Despite these promising findings, there are some general hurdles and limitations for these microRNA-based diagnostic tools. First, all of this data rely on retrospective cohorts and data collections, which are prone to error and selection bias. Therefore, the next logical step has to be a prospective validation and comparison (preferable in independent centralised review laboratories) to the diagnostic gold standard (i.e., the histopathological diagnosis) in a blinded manner. This comparison has to include the assay performance criteria-like specificity and sensitivity, as well as other parameters including cost effectiveness, duration of time from biopsy to diagnosis and applicability in routine diagnostic laboratories (including the necessity of special trained personal and devices). Other problems that are obviously to all gene expression assays (irrespective of whether quantitative PCR, array technology or RNA sequencing is used) are intratumoral and intercellular heterogeneity. Intratumoral heterogeneity is a major cause of misinterpretation of all molecular tests, as the molecular and microRNA profiles significantly differ between different areas of the tumour (Gerlinger *et al*, 2012). On the other hand, the expression profile of tumours is significantly influenced by bystander cells of the tumour stroma and contamination with stromal cells can lead to wrong conclusions and irreproducible microRNA expression results (Kent *et al*, 2014). Both intratumoral heterogeneity and cellular heterogeneity are essential points for the diagnostic process with microRNA-based assays. Alternative methods such as *in situ* hybridisation can offer a real localisation of microRNAs in tumour cells and might overcome some of these limitations, but owing to their rather semiquantitative nature, lack of standardisation and time-consuming procedure, have their own pitfalls.

## MicroRNAs in Cancer Prognosis

The next level of information retrieved by the use of microRNA-based assays is the prediction of the individual risk of tumor progression and clinical endpoints. Traditionally, individual risk stratification and patient counselling mainly rely on clinical and pathological parameters. However, some of these parameters show a high interobserver variability (e.g., tumour grade or Ki-67 staining) and even predictive accuracy of the combination of such prognostic factors to prognostic scores is far from perfect (Pichler *et al*, 2011). Novel laboratory-based or molecular factors including microRNAs to these established prognostic factors and models can significantly increase the predictive ability (Szkandera *et al*, 2014). There are hundreds of published studies proposing the value of different microRNAs as prognostic biomarkers in every different type of cancer. As with many of such prognostic biomarker studies, many of them lack an independent validation and almost all of them are retrospective in their nature. The problems of aforementioned intratumoral and cellular heterogeneity apply also for prognostic biomarkers (Gerlinger *et al*, 2012; Kent *et al*, 2014). For these and other reasons, microRNA-based prognostic assays are far away from approval in clinical routine use and, similar to diagnostic microRNA-based biomarkers, large prospective studies are needed to evaluate their true value in a particular clinical scenario.

Nevertheless, there have been several interesting studies published and some of these microRNA-based prognosticators might warrant further clinical validation. For instance, a very recently published study measured the microRNA profile of colorectal cancer patients in primary tumors compared to metastasis (‘metastatic-signature') and identified 23 microRNAs as differentially expressed. Five of these microRNAs could be validated in a second cohort, in which four of them were downregulated (let-7i, miR-10b, miR-221, and miR-320a) and one was upregulated (miR-885-5p) in liver metastases compared with the primary tumour. Interestingly, low let-7i expression in primary tumour tissue predicted poor prognosis (HR=5.0, 95% CI=1.0–24.4, *P*=0.0479) as well as distant metastasis (OR=5.5, 95% CI=1.1–26.8, *P*=0.0334). High miR-10b expression in primary tumour tissue independently predicted distant metastasis (OR=4.9, 95% CI=1.2–19.7, *P*=0.0248). Furthermore, high serum miR-885-5p expression independently predicted prognosis (HR=2.9, 95% CI=1.1–7.5, *P*=0.0323), lymph node metastases (OR=3.0, 95% CI=1.3–7.2, *P*=0.0116) and distant metastases (OR=3.1, 95% CI=1.0–10.0, *P*=0.0456; Hur *et al*, 2015). The combination of prognostic studies in patient cohorts together with the consequently experimental proof and explanation of biological functions and molecular interactions of microRNAs can substantiate the prognostic significance of a given microRNA. In line with this, a very recently published study by Ling *et al* (2015) proposes that miR-224 is a negative prognostic factor in colorectal cancer patients. Multiple cohorts were used to demonstrate the prognostic value of miR-224 and by using *in vitro* and *in vivo* models, the authors experimentally confirmed miR-224 to promote tumour metastases. Similarly, miR-200a has been reported as prognostic relevant in a screening cohort of 110 colorectal cancer patients and has been validated in independent samples of the Cancer Genome Atlas. The authors of this study substantiate the prognostic value by showing experimental data about the involvement of miR-200a in epithelial–mesenchymal transition, a fundamental process for cancer metastases in colorectal cancer (Pichler *et al*, 2014). Recently, a large study in B-cell lymphoma patients demonstrated well-defined microRNA signatures for normal B cells as well as subsets of lymphoma cells. High expression levels of miR-155 were identified as significantly associated with rituximab plus cyclophosphamide, doxorubicin, vincristine and prednisone (R-CHOP) treatment failure (Iqbal *et al*, 2015). Studies like these generate data for potential biomarkers but might also be fundamental to discover druggable microRNAs for cancer therapy.

## MicroRNAs in Cancer Therapy

Of all microRNA-based applications in cancer medicine, the therapeutic potential of microRNAs might be the most promising and challenging path. On the one hand, microRNAs interact with multiple targets including several mRNAs of the same signalling pathway, which might potentiate the efficacy of microRNA-based drugs. However, having several potential interactors will also carry risk of off target effects resulting in frequently occurring and severe adverse events in other organs. Numerous *in vitro* and *in vivo* studies have demonstrated efficacy for microRNAs to interfere with all hallmarks of cancer ultimately resulting in tumour regression and cancer cell death. The mode of action of microRNA-based drugs can either rely on restoring their loss of function (for tumour suppressive microRNAs) or inhibiting their gain of function (for oncomiRs). One innovative approach has been published by Nishimura and colleagues, where the authors presented data about a double targeting strategy by combining a microRNA together with a short interfering RNA (siRNA). The authors used a siRNA against the EphA2 oncogene in a preclinical model of ovarian cancer and boosted the antitumour effects by addition of miR-520-3d, which synergistically inhibited the EphA2 expression in cancer cells. Nishimura *et al.* used 1,2-dioleoyl-sn-glycero-3-phosphatidylcholine nanoliposomes loaded with miR-520d-3p and EphA2 siRNA and clearly demonstrated a synergy of this combined treatment to shrink the tumours, which might have broad implications for innovative gene-silencing therapies in clinical trials (Nishimura *et al*, 2013). One of the most advanced microRNA-based therapeutic candidates currently evaluated in clinical trials is MRX34 (Mirna Therapeutics, TX, USA), a miR-34 mimetics that restores the function miR-34 in cancer cells. MiR-34 is frequently downregulated in human cancers and acts as a tumour suppressive microRNA. Most recent data of an ongoing multicenter phase I clinical trial protocol for patients with liver cancer and liver metastases of other cancers have been presented in April 2015 at the Annual Meeting of the American Association for Cancer Research (AACR). Interims safety data indicate a manageable profile of side effects and in white blood cells of patients the repression of expression of several potential miR-34 target oncogenes could be proven (Hong *et al*, oral presentation at AACR 2015). Another, though preclinical work, presented recently at the AACR includes data derived from an *in vivo* study in nonsquamous lung cancer demonstrating that miR-34 directly represses the checkpoint signalling molecule PD-L1 (programmed death ligand 1) and that MRX34 treatment leads to an increase in active tumour-infiltrating immune cells (CD8+) and a decrease in CD8+PD1+ tumour-infiltrating immune cells (Cortez *et al*, oral presentation at AACR 2015, abstract # 2875). Hopefully within the next several months we will receive more details about efficacy of this exciting first-in-class clinical trial. Besides the use of microRNAs as drug candidates themselves there is another emerging field related to microRNAs in cancer therapy. MicroRNA-based predictive biomarkers hold promise to inform about the probability of response rates of other (microRNA-unrelated) cancer drugs (Stiegelbauer *et al*, 2014). A representative example is the value of microRNAs for the prediction of epidermal growth factor receptor-directed therapies (e.g., cetuximab). In colorectal cancer patients, a single-nucleotide polymorphism in the let-7 binding site of the KRAS gene, has been proposed to predict the tumour responsiveness (a particular allele combination resulted in overall response rate of a 42% compared with a 9%) in cetuximab-treated patients (Zhang *et al*, 2011). Another example is miR-212, which has been involved in cetuximab-resistant cancer cells of head and neck carcinoma by directly regulating heparin-binding EGF-like growth factor (Hatakeyama *et al*, 2010). Table 1 summarises microRNAs with important roles in cancer diagnosis, prognosis or prediction of response to treatment. Taken together, all these directions are innovative and promising, but the proof of concept in preclinical models has to move forward and successfully pass confirmation in prospective clinical trials. The next up-coming years will verify whether these small molecules will help to substantially improve cancer diagnosis and treatment or just represent another small piece of the large puzzle.

## Figures and Tables

**Figure 1:**
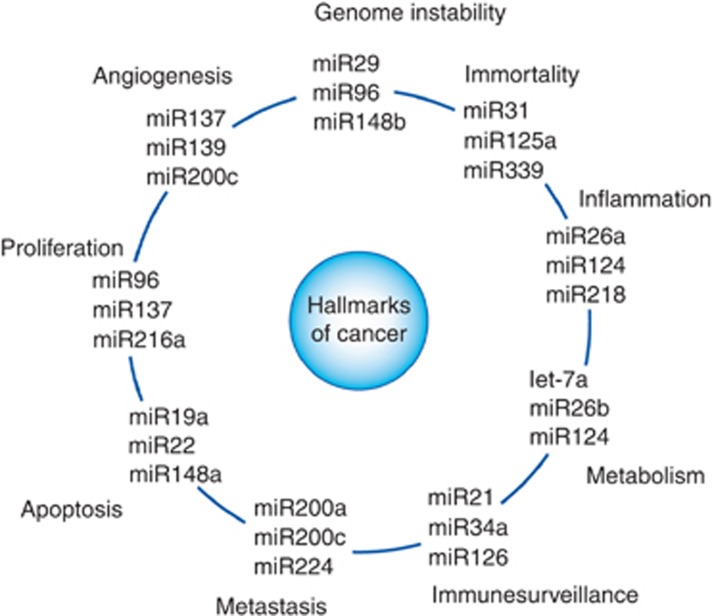
**Schematic illustration of the association between microRNAs and the hallmarks of cancer.** Each hallmark shows three examples of microRNAs that influence the particular cellular function in certain types of cancer. Of note, some microRNAs influence more than one hallmark indicating to the multiple pathways regulated by them.

**Table 1:** Examples of important studies that implicated microRNAs in cancer diagnosis, prognosis and prediction of response to cancer drugs

**Purpose**	**MicroRNA(s)**	**Type of cancer**	**Sample**	**Authors**
Early detection	13 microRNA panel (‘miR-Test')	Lung cancer	Serum	Montani *et al*, 2015
Diagnosis	24 microRNA panel	Kidney cancer	Tissue	Spector *et al*, 2013
Diagnosis	3 microRNA panel (miR21, miR31 and miR210)	Lung cancer	Sputum	Xing *et al*, 2015
Diagnosis	3 microRNA panel (miR200c, -93-3p, -192)	Mesothelioma	Tissue	Benjamin *et al*, 2010
Diagnosis	8 microRNA panel (miR106a, -125a-5p, -129-3p, -205, -21, -29b, -375, -7)	Lung cancer	Tissue	Gilad *et al*, 2012
Diagnosis	64 microRNA panel	Cancer of unknown primary	Tissue	Meiri *et al*, 2012
Diagnosis	48 microRNA panel	Cancer of unknown primary	Tissue	Rosenfeld *et al*, 2008
Prognosis	miR224	Colorectal cancer	Tissue	Ling *et al*, 2015
Prognosis	miR200a	Colorectal cancer	Tissue	Pichler *et al*, 2014
Prognosis	let-7i, miR-10b, miR-885-5p	Colorectal cancer	Tissue	Hur *et al*, 2015
Prognosis	miR155	Lymphoma	Cells	Iqbal *et al*, 2015
Prediction	miR212	Head and neck cancer	Tissue	Hatakeyama *et al*, 2010
Prediction	let7-binding site in KRAS gene	Colorectal cancer	Tissue or blood	Zhang *et al*, 2011
